# *Aonchotheca* (Nematoda: Capillariidae) is validated as a separated genus from *Capillaria* by both mitochondrial and nuclear ribosomal DNA

**DOI:** 10.1186/s13071-022-05609-9

**Published:** 2022-12-30

**Authors:** Yuan-Ping Deng, Suleman, Xue-Ling Zhang, Rong Li, Le-Yan Li, Yi-Tian Fu, Guo-Hua Liu, Chaoqun Yao

**Affiliations:** 1 Research Center for Parasites and Vectors, College of Veterinary Medicine, Hunan Agricultural University, Changsha, 410128 Hunan China; 2 Department of Zoology, University of Swabi, Swabi, 23561 Khyber Pakhtunkhwa Pakistan; 3 Department of Biomedical Sciences and One Health Center for Zoonoses and Tropical Veterinary Medicine, Ross University School of Veterinary Medicine, Basseterre, Saint Kitts and Nevis

**Keywords:** *Aonchotheca putorii*, Capillariidae, Mitogenome, Genetic distance, 18S rRNA, Phylogenetic relationship

## Abstract

**Background:**

The family Capillariidae is a group of thread-like nematodes of 27 genera and over 300 species that infect a great variety of hosts including humans. Among these, some taxa such as the genus *Aonchotheca* have remained controversial regarding their systematic status for decades. The aim of the current study was to verify *Aonchotheca*’s systemic status and to further determine whether it is a distinct genus from *Capillaria* using molecular and phylogenetic analyses.

**Results:**

We sequenced the mitochondrial (mt) genome and nuclear small subunit (18S) rRNA gene of *Aonchotheca putorii*, a representative species of the genus, and investigated its systematic status in Trichinellida using maximum likelihood and Bayesian inference. The differences in amino acid sequences of 13 protein-coding genes were 12.69–67.35% among *Aonchotheca*, *Capillaria*, *Eucoleus*, and *Pseudocapillaria* with *cox*1 (12.69%) and *atp*8 (67.35%) as the most and the least conserved gene, respectively, and the difference of two mt rRNAs was 18.61–34.15%. Phylogenetic analyses of the complete mt genome and 18S rRNAs unequivocally showed that *Aonchotheca* was a distinct genus from *Capillaria*.

**Conclusions:**

Large difference exists among *Aonchotheca*, *Capillaria*, *Eucoleus,* and *Pseudocapillarias*. *Aonchotheca putorii* is the first species in the genus *Aonchotheca* for which a complete mitogenome has been sequenced. These data are useful for phylogenetics, systematics and the evolution of Capillariidae.

**Graphical Abstract:**

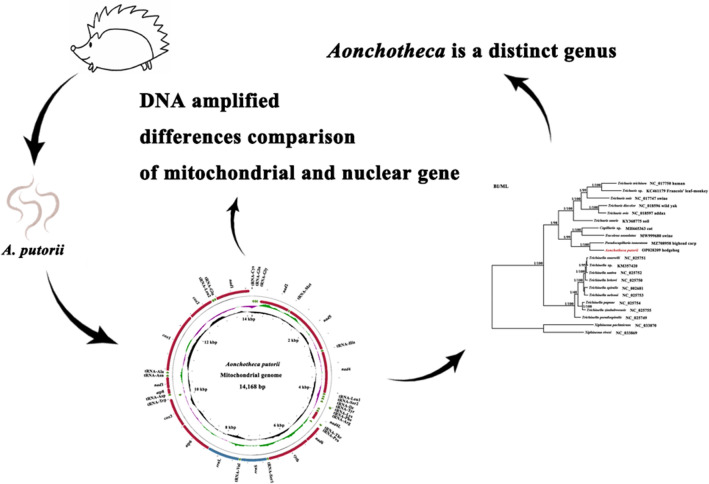

**Supplementary Information:**

The online version contains supplementary material available at 10.1186/s13071-022-05609-9.

## Background

Capillariidae (Railliet, 1915) is a family of thread-like nematodes traditionally differentiated from others by the characteristic esophagus, male caudal end, and unique morphology of eggs [1–3]. So far, over 300 species have been recorded worldwide in almost all groups of vertebrate hosts such as fishes, reptiles, amphibians, birds, mammals, and humans [1, 4, 5]. Capillariids mainly parasitize the internal organs of hosts such as the respiratory and digestive tract, esophagus, stomach, and liver, severely damaging them [1, 3]. However, the identification of Capillariidae to species or even genus by morphology alone is one of the most complicated and challenging tasks among all nematodes. Consequently, controversies on some taxon remain until now [4, 6, 7]. Some taxonomic experts have proposed several new genera and synonymies of capillariids and suggested reclassifying this group [1, 3].

The use of DNA-based molecular techniques is one of the recent advancements for the accurate identification and understanding of nematode evolutionary relationships [8, 9]. The nuclear small subunit of the ribosomal gene (18S rRNA) has been frequently used to analyze phylogenetic relationships among nematodes at different taxonomic levels, especially of some representative capillariids [5, 10–14]. Genome-based phylogenetic analyses such as mitochondrial (mt) genome (mitogenome) are increasingly utilized by the taxonomist for a better understanding of nematodes’ relationships at various taxonomic levels and/or judgment of the discordances and congruences with morphology-based and nuclear rDNA-based phylogenies due to the shortcomings of 18S rRNA [15, 16]. Within the family Capillariidae, the currently available mitogenomes are representatives of the genus *Capillaria* Zeder, 1800 (MH665363), *Eucoleus* Dujardin, 1845 (NC_056391), and *Pseudocapillaria* Freitas, 1959 (MZ708958).

Members of the genus *Aonchotheca* López-Neyra, 1947, are prevalent in many geographical regions and mainly infect the stomach and intestines of wild carnivores, especially mustelids [17]. They utilize earthworms as a reservoir to enhance their infection to the definitive hosts [18]. The taxonomic status of *Aonchotheca putorii* (Rudolphi, 1819) López-Neyra, 1947, within Capillariidae has also been revised several times, and it is often considered a species of the genus *Capillaria* [18]. Intra-specific morphometric variations among capillariids have been frequently observed because of their phenotypic plasticity, physiological and/or immunological effects of different hosts or even processing techniques in different laboratories [19]. Tamaru et al. observed significant nucleotide variation in the 18S rRNA sequence among specimens of *A. putorii* collected from various wild carnivores in Japan [19]. In contrast, Kołodziej-Sobocinska et al. did not observe significant genetic divergence in it among *A. putorii* worms collected from either same or different hosts at distant geographical localizations [20]. Hence, the two aims of the current study were to (1) analyze the genetic divergence in the 18S rRNA sequence of *A. putorii* from different hosts and locations and (2) verify the status of *Aonchotheca* by comparing mt coding regions and analyzing phylogenetic relationships within the family Capillariidae.

## Materials and methods

### Parasites and molecular identification

Adult parasites were collected from the small intestine of dead hedgehogs (*Erinaceus europaeus* Linnaeus, 1758) originated from Beijing, China. The specimens were intensively washed in ultrapure water and physiological saline solution, and then stored in 75% ethanol. They were morphologically identified as capillariid nematodes with the presence of precloacal caudal alae in male worms [5]. For molecular identification, the total genomic DNA was extracted from several worms using QIAamp^®^ DNA Micro Kit (Qiagen, Hilden, Germany) per the manufacturer’s instruction. Polymerase chain reaction (PCR) was applied to amplify mitochondrial (mt) gene *cox*1 using the primers JB3-JB4.5 as previously described [21] and nuclear 18S rRNA by the newly designed ones (F: 5ʹ-TTG GTG CGT TCG GTT CGC TGT T-3ʹ; R: 5ʹ-CCA AGC GAG CAG CAT CAG TCC A-3ʹ). PCR was carried out in BIO-RAD T100™ thermal cycler (Bio-Rad, Hercules, CA, USA) using the following conditions: 95 ℃ 1 min, followed by 37 cycles of 98 ℃ 10 s, 60 ℃ 30 s and 72 ℃ 1 min, with a final extension at 72 ℃ 8 min. The PCR amplicons were purified using NucleoSpin^®^ Gel and PCR Clean-up (Takara Bio USA, Inc.) and sent to BGI Co., Ltd. (Shenzhen, China) for direct sequencing in both directions. The DNA sequences were assembled by DNAMAN v6.0 (Lynnon Biosoft, USA). These sequences were then used to BLAST search GenBank to confirm their identities and species identification of the worms of DNA origin.

### Sequencing and assembling mitochondrial genome

Total genomic DNA was fragmented to about 350 bp. The DNA libraries were sequenced using Illumina Hiseq 6000 platform (Novogene Co. Ltd. Tianjin, China). The raw sequences were in FASTQ format. Clean DNA sequences were obtained by removing adapter sequences, low-quality bases (Phred quality < 5), or uncertain reads with repetitive “N” bases. The partial *cox*1 sequence derived from PCR amplicons as described earlier was then used as the initial reference to assemble the complete mitogenome sequence of *A. putorii* using Geneious Prime v.2022.0.1 [22] with the following parameters: (i) minimum overlap within the range of 150–200 bp; (ii) minimum overlap identity of 99%; (iii) maximum gap of 5 bp. The complete circular mitogenome of the roundworm was then verified by PCR with primers as listed (Additional files 1 and 2: Table S1 and Figure S1).

### Annotation of mitochondrial genome

The webserver MITOs was used for the preliminary annotation of 37 genes in the above-assembled mitogenome [23]. Initiation/termination codons and gene boundaries for all 13 protein-coding genes (PCGs) were identified by Open Reading Frame (ORF) Finder of NCBI (https://www.ncbi.nlm.nih.gov/orffinder/). Locations and borders of two rRNAs (*rrn*L and *rrn*S) were recognized based on Tandem [24] and aligned capillariid sequences. Similarity, sequences, directions, and the secondary structures of 22 transfer RNA genes (tRNAs) were further verified by tRNAscan-SE 2.0 with a cutoff score of 1.0 [25]. MEGA v11.0 was used to verify accuracies of 13 PCGs and infer 13 amino acid sequences, codon usage and relative synonymous codon usage (RSCU) [26]. The values of the non-synonymous (Ka)/synonymous (Ks) substitution ratios were calculated with DnaSP v5 to understand the selective stress in the evolution of the family Capillariidae.

### Phylogenetic analyses

The 18 complete mitogenomes in the order Trichinellida available in GenBank were all included in phylogenetic analyses. To better understand the evolution and phylogenetic relationships of Trichinellida lineage, we used *Xiphinema pachtaicum* Tulaganov, 1938 (GenBank no. NC_033870) and *X. rivesi* Dalmaso, 1969 (GenBank no. NC_033869) as outgroups [27] (Table 1), as Dorylaimida evolves more rapidly in their mitogenome than Trichinellida within the class Enoplea [10]. The aligned amino acid sequences by MAFFT online server with “L-INS-I” [28] were concatenated into a single alignment database and uploaded to Gbolcks 0.91b [29] to exclude ambiguity and identify more conserved blocks by selecting the option for “more stringent.” Bayesian inference (BI) and maximum likelihood (ML) were applied to phylogenetic analyses. Using Mrbayes 3.2 [30] with default model “JC69,” BI tree was constructed with four independent Markov chains by analyzing 1,000,000 generations and sampling tree every 100 generations. Based on ModelFinder in IQTree v.2.1.3 and ProtTest 3.4.2 [31, 32], “mtZOA + F + R5” and “MtArt + I + G + F” were selected as the most suitable models for ML analysis in IQTree v.2.1.3 and PhyML 3.1, respectively. Additionally, available 18S rRNA sequences within the family Capillariidae were used to analyze nuclear phylogenetic relationships and genetic distances. The 18S rRNA sequences were aligned to a single database and used to constructed phylogenetic analyses based on ML (best model: GTR + I + G) and BI (best model: GTR) with the same steps as above. The alignment database was also applied to generate genetic distances of 18S rRNA using MEGA v11.0 [26].

**Table 1:** Mitochondrial genome sequences of Trichinellida nematodes available prior to the present study were used for phylogenetic analyses

Family	Species	Size (bp)	GenBank accession no.
Trichuridae	*Trichuris suis*	14,436	NC_017747
	*Trichuris trichiura*	14,046	NC_017750
	*Trichuris ovis*	13,946	NC_018597
	*Trichuris discolor*	13,904	NC_018596
	*Trichuris muris*	14,095	KY368775
	*Trichuris* sp.	14,147	KC461179
Trichinellidae	*Trichinella spiralis*	16,706	NC_002681
	*Trichinella nelsoni*	15,278	NC_025753
	*Trichinella nativa*	14,077	NC_025752
	*Trichinella murrelli*	16,592	NC_025751
	*Trichinella britovi*	16,421	NC_025750
	*Trichinella papuae*	17,326	NC_025754
	*Trichinella pseudospiralis*	17,667	NC_025749
	*Trichinella zimbabwensis*	14,244	NC_025755
	*Trichinella* sp.	16,308	KM357420
Capillariidae	*Capillaria* sp.	13,624	MH665363
	*Eucoleus annulatus*	14,118	MW999680
	*Pseudocapillaria tomentosa*	14,062	MZ708958

## Results and discussion

### First molecular identification of *A. putorii* in China

The mt *cox*1 sequence of PCR amplicon of 652 bp (GenBank accession no: OP363931) using primer pair JB3-JB4.5 as previously described [21] showed only 81.7% identities to GenBank no. MH665361 by BLAST search, which was a *Capillaria* sp. This low identity indicates the parasitic worm may be one member of the family Capillariidae and relate to *Capillaria* sp. species. We then PCR amplified and sequenced 18S rRNA of 1813 bp. The newly obtained 18S rRNA sequence (GenBank accession no: OP028951) of this nematode showed 100% identity to GenBank no. LC052349 of *A. putorii*. Therefore, the nematodes recovered from the small intestine of hedgehogs in Beijing, China, are identified as *A. putorii*, which is the first report in the country to our knowledge. *Aonchotheca. putorii* has been found in a wide range of geographical locations and several mammalian hosts including minks, weasels, martens, racoons, and even the domestic cats [17].

### Divergency of 18S rRNA gene in the family Capillariidae

We then further analyzed the genetic divergence of 18S rRNA of 19 members in the family Capillariidae (Additional file 1: Table S2), which ranged from 0 to 14.1%. The 18S rRNA of *Capillaria spinulosa* (Linstow, 1890) was the most variable (10.9–14.1%) (Additional file 1: Table S2). Kołodziej-Sobocinska et al. reported no significant genetic divergence of 18S rRNA genes was observed in *A*. *putorii* collected from different geographical distributions and various hosts [20]. However, Tamaru et al. found significant nucleotide variation of 18S rRNA genes among *A*. *putorii* collected from various animals [19]. Our result showed clear divergence in 18S rRNA sequences of worms recovered from Japan, America, and China. Specifically, comparing currently available 500 bp of 18S rRNA isolated from American *A. putorii* with those from China and Japan, up to 4.00% divergency was found. Furthermore, up to 8.94% (31 nucleotide substitutions) difference was found between Japanese and Chinese isolates (Additional file 1: Table S3).

### First complete mt genome of *A. putorii*

**Fig. 1:**
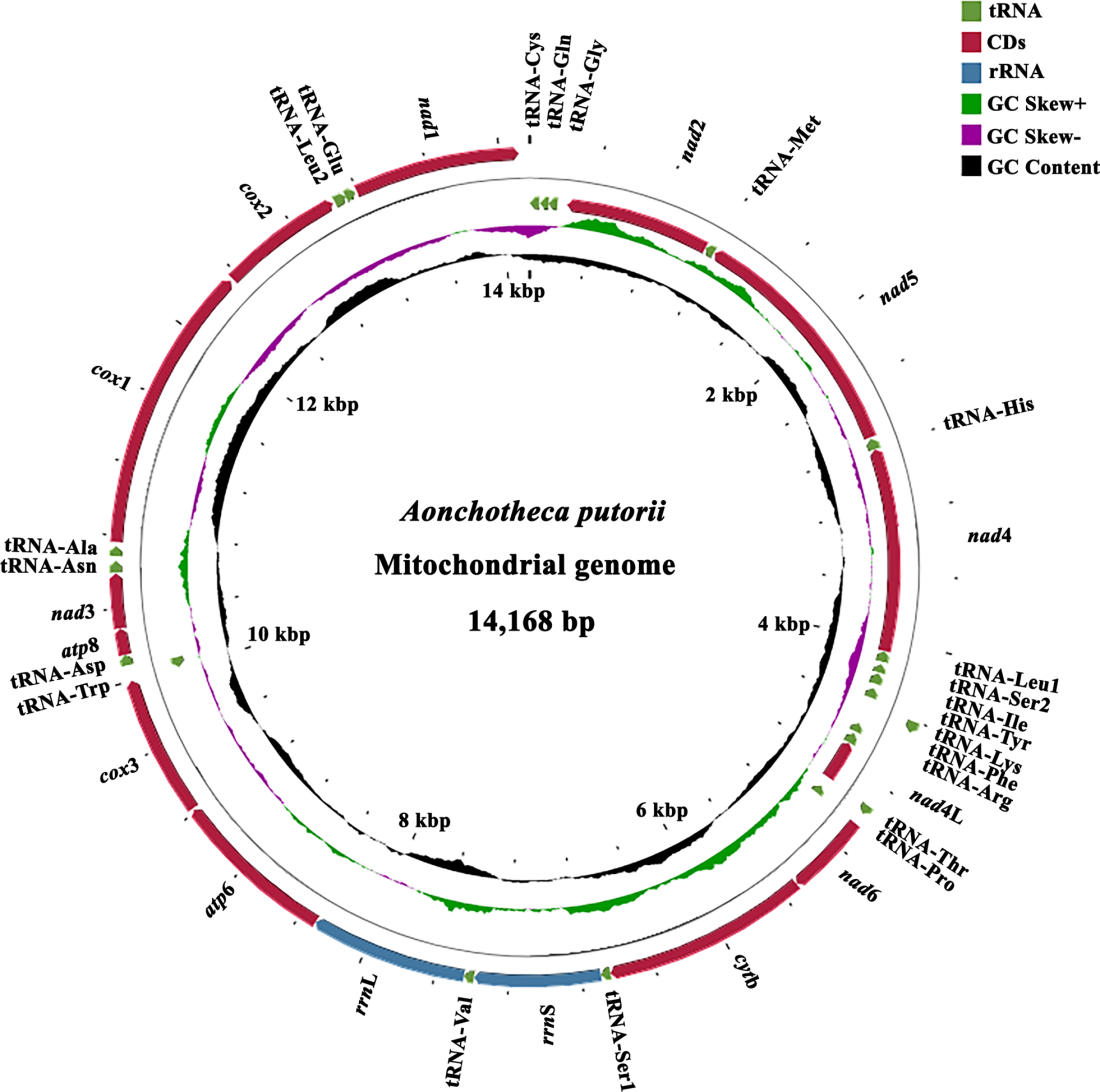
The organization of the complete mitochondrial genome of *Aonchotheca putorii* (Rudolphi, 1819) López-Neyra, 1947. The scale is approximate. Different colors represent different elements; the directions of arrows represent the directions of gene transcriptions; the height of peaks represent the value of GC skew + , GC skew- and GC content. *atp*6 and *atp*8 ATP synthase F0 subunits 6 and 8, *cyt*b cytochrome b, *cox*1‑3 cytochrome *c* oxidase subunits 1–3, *nad*1‑6 and *nad*4L NADH dehydrogenase subunits 1–6 and 4L, *rrn*S and *rrn*L small and large subunits of ribosomal RNA

We then tried to decode the whole mt genome of *A. putorii*. Due to the lack of mt genome of closely related species, we took an unusual strategy using the newly *cox*1 DNA sequence just mentioned and next generation sequencing technique as outlined earlier. The complete mitogenome of *A. putorii* was 14,168 bp in length and had been uploaded into GenBank with the accession number OP028209. It comprised 37 genes (Table 2, Fig. 1) including 13 PCGs (*atp*6, *atp*8, *cox*1-*cox*3, *cyt*b, *nad*1-*nad*6, and *nad*4L), 2 rRNAs (12S rRNA and 16S rRNA) and 22 tRNAs (Fig. 1). Its genetic arrangement was consistent with those of published *Eucoleus annulatus* (Molin, 1858) López-Neyra, 1946 (MW999680) [33], and *Pseudocapillaria tomentosa* (Dujardin, 1843) Lomakin and Trofimenko, 1982 (MZ708958), but different from that of *Capillaria* sp. (MH665363). The findings indicated at least one inversion between *Aonchotheca* − *Eucoleus* − *Pseudocapillaria* species and *Capillaria* sp., though four tRNAs (tRNA-Gly, tRNA-Tyr, tRNA-Cys and tRNA-His) were lacking in the *Capillaria* sp.. The tRNA-Gln was found on the L-strand between tRNA-Cys and tRNA-Gly in *A. putorii*, *E. annulatus,* and *P*. *tomentosa*, while it was located between *nad*5 and *nad*4 in *Capillaria* sp. (not shown).

**Table 2:** The organization of the mt genome of *Aonchotheca putorii* from Beijing, China

Gene/region	Strand^a^	Positions	Size (bp)	Number of aa^b^	Ini/Ter^c^ codons	Anticodons
tRNA-Cys (C)	L	59-1	59			GCA
tRNA-Gln (Q)	L	118-65	54			TTG
tRNA-Gly (G)	L	172-119	54			TCC
*nad*2	L	1134-235	900	299	ATT/TAA	
tRNA-Met (M)	L	1195-1135	61			CAT
*nad*5	L	2721-1201	1521	506	ATA/TAA	
tRNA-His (H)	L	2801-2731	71			GTG
*nad*4	L	4064-2802	1263	420	ATA/TAA	
tRNA-Leu^CUN^ (L_1_)	L	4132-4065	68			TAG
tRNA-Ser^UCN^ (S_2_)	L	4203-4150	54			TGA
tRNA-Ile (I)	L	4275-4210	66			GAT
tRNA-Tyr (Y)	L	4364-4304	61			GTA
tRNA-Lys (K)	H	4405-4467	63			TTT
tRNA-Phe (F)	L	4595-4540	56			GAA
tRNA-Arg (R)	L	4675-4610	66			TCG
*nad*4L	L	4925-4677	249	82	ATA/TAA	
tRNA-Thr (T)	H	4927-4981	55			TGT
tRNA-Pro (P)	L	5044-4991	54			TGG
*nad*6	H	5046-5504	459	152	ATT/TAA	
*cyt*b	H	5507-6642	1136	378	ATG/TA	
tRNA-Ser^AGN^ (S_1_)	H	6643-6695	53			TCT
*rrn*S	H	6696-7388	693			
tRNA-Val (V)	H	7389-7444	56			TAC
*rrn*L	H	7445-8308	864			
*atp*6	H	8309-9217	909	302	ATA/TAA	
*cox*3	H	9226-9999	774	257	ATG/TAA	
tRNA-Trp (W)	L	10064-10003	62			TCA
tRNA-Asp (D)	H	10088-10146	59			GTC
*atp*8	H	10147-10292	146	48	ATT/TA	
*nad*3	H	10293-10593	301	100	ATT/T	
tRNA-Asn (N)	H	10653-10714	62			GTT
tRNA-Ala (A)	H	10742-10795	54			TGC
*cox*1	H	10797-12347	1584	527	ATT/TAA	
*cox*2	H	12365-13048	684	227	ATA/TAA	
tRNA-Leu^UUR^ (L_2_)	H	13063-13129	67			TAA
tRNA-Glu (E)	H	13129-13185	57			TTC
*nad*1	H	13196-14107	912	303	ATT/TAA	

*atp*6 and *atp*8 ATP synthase F0 subunits 6 and 8, *cyt*b cytochrome b, *cox*1‑3 cytochrome *c* oxidase subunits 1–3, *nad*1‑6 and *nad*4L NADH dehydrogenase subunits 1–6 and 4L, *rrn*S and *rrn*L small and large subunits of ribosomal RNA

^a^L = L-stand; H = H-stand

^b^The inferred length of amino acid (aa) sequence of 13 protein-coding genes

^c^The initiation and termination codons

Twenty-five intergenic regions ranging from 1 to 72 bp in length were found scattered throughout the mitogenome. The highest AT content of 84.8% was in intergenic regions, and AT-skew in these regions was positive while the value of GC-skew in them was “0” (the number of G bases was roughly equal to C bases, Table 3). The mitogenomes of nematodes usually contain two non-coding regions (NCRs) with different sizes [15, 33, 34]. The length of AT-rich was 137 bp in *Necator americanus* Stiles, 1902 [35], 886 bp in *Ascaris suum* Goeze, 1782 [36], and approximately 7 kb in *Hoplolaimus columbus* Sher, 1963 [37]. As far as members of the family Capillariidae were concerned, no AT-rich region was found in the mitogenome of *A. putorii*, but it was 306 bp, 121 bp and 99 bp in *E. annulatus* [33], *Capillaria* sp. (MH665363) and *P. tomentosa* (MZ708958), respectively.

**Table 3:** Nucleotide composition and skews of *Aonchotheca putorii* mitochondrial genome

Gene	Nucleotide frequency				A + T (%)	AT-skew	GC-skew
	A (%)	G (%)	T (%)	C (%)			
*atp*6	34.6	11.2	46.2	8.0	80.8	− 0.144	0.166
*atp*8	36.3	11.0	46.6	6.1	82.9	− 0.124	0.280
*cox*1	32.7	15.1	39.8	12.4	72.5	− 0.096	0.096
*cox*2	36.7	14.5	36.3	12.5	73.0	0.006	0.070
*cox*3	31.8	13.8	43.7	10.7	75.5	− 0.158	0.126
*cyt*b	27.1	17.6	46.8	8.5	73.9	− 0.267	0.345
*nad*1	36.3	12.5	40.0	11.2	76.3	− 0.049	0.056
*nad*2	39.9	6.0	41.8	12.3	81.7	− 0.023	− 0.345
*nad*3	30.3	10.7	50.3	8.7	80.6	− 0.248	0.103
*nad*4	41.1	8.4	40.1	10.5	81.2	0.013	0.109
*nad*4L	42.6	10.0	37.0	10.4	79.6	0.071	− 0.020
*nad*5	39.3	8.7	38.7	13.3	78.0	0.007	− 0.206
*nad*6	26.8	10.9	55.6	6.7	82.4	− 0.349	0.235
*rrn*S	34.9	14.4	41.4	9.3	73.3	− 0.085	0.220
*rrn*L	41.1	11.1	40.4	7.4	81.5	0.009	0.200
22 tRNAs	36.5	11.0	42.7	9.8	79.2	− 0.079	0.055
NCR	43.5	7.8	40.9	7.8	84.4	0.031	0
Total	35.8	12.7	42.2	9.3	78.0	− 0.082	− 0.157

*Aonchotheca putorii* mitogenome was A-T biased; they accounted for 78.0% (Table 3), which is consistent with other capillariid nematodes [33]. Furthermore, T base (42.2%) was the most frequent and C (9.3%) the least. Both AT-skew and GC-skew included negative and positive values, ranging from − 0.349 (*nad*6) to 0.031 (NCRs) and from − 0.345 (*nad*2) to 0.345 (*cyt*b), respectively.

### Characteristics of protein-coding genes

Aligning with available sequences of capillariid nematodes (GenBank no. MZ708958, NC_056391 and MH665363), the boundaries of each PCG were determined, and the initiation/termination codons and the directions of translation were identified. For *A. putorii*, the longest PCG was *cox*1 (1584 bp), followed by *nad*5 (1521 bp). The rest were *nad*4 (1,263 bp), *cyt*b (1136 bp), *nad*1 (912 bp), *atp*6 (909 bp), *nad*2 (900 bp), *cox*3 (774 bp), *cox*2 (684 bp), *nad*6 (459 bp), *nad*3 (301 bp), *nad*4L (249 bp) and *atp*8 (146 bp).

ATN was exclusively used as an initiation codon in all PCGs where N was T, A or G. ATT was the most frequent initiation codon used in six PCGs (*nad*2, *nad*6, *atp*8, *nad*3, *cox*1 and *nad*1), followed by ATA in five (for *nad*5, *nad*4, *nad*4L, *atp*6 and *cox*2). The least favored start codon was ATG used only in *cyt*b and *cox*3. The complete termination codon TAA was the most common one, which was used in all PCGs except *cyt*b, *atp*8, and *nad*3, in which incomplete stop codons TA and T were used, respectively. Start codons ATT, ATA and ATG were shared among those species, while TTG was only used in *E. annulatus* and *Capillaria* sp., and ATC was unique in *P. tomentosa*. Complete termination codon TAA was prevalent among those species, but TAG was used in *E. annulatus*, *Capillaria* sp. and *P*. *tomentosa* excluding *A. putorii*.

The GC- and AT-skews for mitogenome are calculated as a measure of the compositional asymmetry and effective assistant of replication [38, 39]. For the *A. putorii* mitogenome, the AT- and GC-skews were − 0.082 and − 0.157, respectively, which showed no clear trend of A or T bases, but a significant use of C bases compared with other nematodes [40]. Similar bases trend also showed in PCGs. AT and GC-skews of 13 PCGs were from − 0.349 (*nad*6) to 0.071 (*nad*4L) and from − 0.345 (*nad*2) to 0.345 (*cyt*b) (Table 3), indicating a trend of A bases among those PCGs that would have an impact on RSCU. RSCU represents an intuitive reflection of the use of codon bias, and a higher RSCU indicates a higher codon usage bias. Within codons encoding amino acids, there was an obvious bias of T- or A-rich codons. For example, Leu was encoded by TTA, TTG, CTT, CTC, CTA and CTG, but the RSCU value of TTA was 3.91 higher than that with C and G residues (Table 4).

**Table 4:** Amino acid frequency and relative synonymous codon usage of *Aonchotheca putorii* mitochondrial protein-coding genes

Amino acid	Codon	Number	RSCU (%)	Amino acid	Codon	Number	RSCU (%)
Phe (F)	TTT	307	1.76	Tyr (Y)	TAT	134	1.71
Phe (F)	TTC	41	0.24	Tyr (Y)	TAC	23	0.29
Leu (L)	TTA	317	3.91	Stop (*)	TAA	10	2
Leu (L)	TTG	67	0.83	Stop (*)	TAG	0	0
Leu (L)	CTT	22	0.27	His (H)	CAT	41	1.49
Leu (L)	CTC	6	0.07	His (H)	CAC	14	0.51
Leu (L)	CTA	67	0.83	Gln (Q)	CAA	47	1.84
Leu (L)	CTG	8	0.1	Gln (Q)	CAG	4	0.16
Ile (I)	ATT	338	1.76	Asn (N)	AAT	164	1.62
Ile (I)	ATC	46	0.24	Asn (N)	AAC	38	0.38
Met (M)	ATA	341	1.74	Lys (K)	AAA	119	1.82
Met (M)	ATG	52	0.26	Lys (K)	AAG	12	0.18
Val (V)	GTT	74	1.64	Asp (D)	GAT	48	1.71
Val (V)	GTC	12	0.27	Asp (D)	GAC	8	0.29
Val (V)	GTA	82	1.82	Glu (E)	GAA	75	1.85
Val (V)	GTG	12	0.27	Glu (E)	GAG	6	0.15
Ser (S)	TCT	95	1.95	Cys (C)	TGT	28	1.47
Ser (S)	TCC	13	0.27	Cys (C)	TGC	10	0.53
Ser (S)	TCA	106	2.18	Trp (W)	TGA	104	1.81
Ser (S)	TCG	9	0.19	Trp (W)	TGG	11	0.19
Pro (P)	CCT	38	1.49	Arg (R)	CGT	17	1.48
Pro (P)	CCC	3	0.12	Arg (R)	CGC	0	0
Pro (P)	CCA	56	2.2	Arg (R)	CGA	26	2.26
Pro (P)	CCG	5	0.2	Arg (R)	CGG	3	0.26
Thr (T)	ACT	64	1.71	Ser (S)	AGT	47	0.97
Thr (T)	ACC	15	0.4	Ser (S)	AGC	1	0.02
Thr (T)	ACA	65	1.73	Ser (S)	AGA	114	2.34
Thr (T)	ACG	6	0.16	Ser (S)	AGG	4	0.08
Ala (A)	GCT	38	1.63	Gly (G)	GGT	46	1.29
Ala (A)	GCC	15	0.65	Gly (G)	GGC	5	0.14
Ala (A)	GCA	36	1.55	Gly (G)	GGA	80	2.24
Ala (A)	GCG	4	0.17	Gly (G)	GGG	12	0.34

Excluding abbreviated stop codons (TA and T)

*Stop* stop codon

**Fig. 2:**
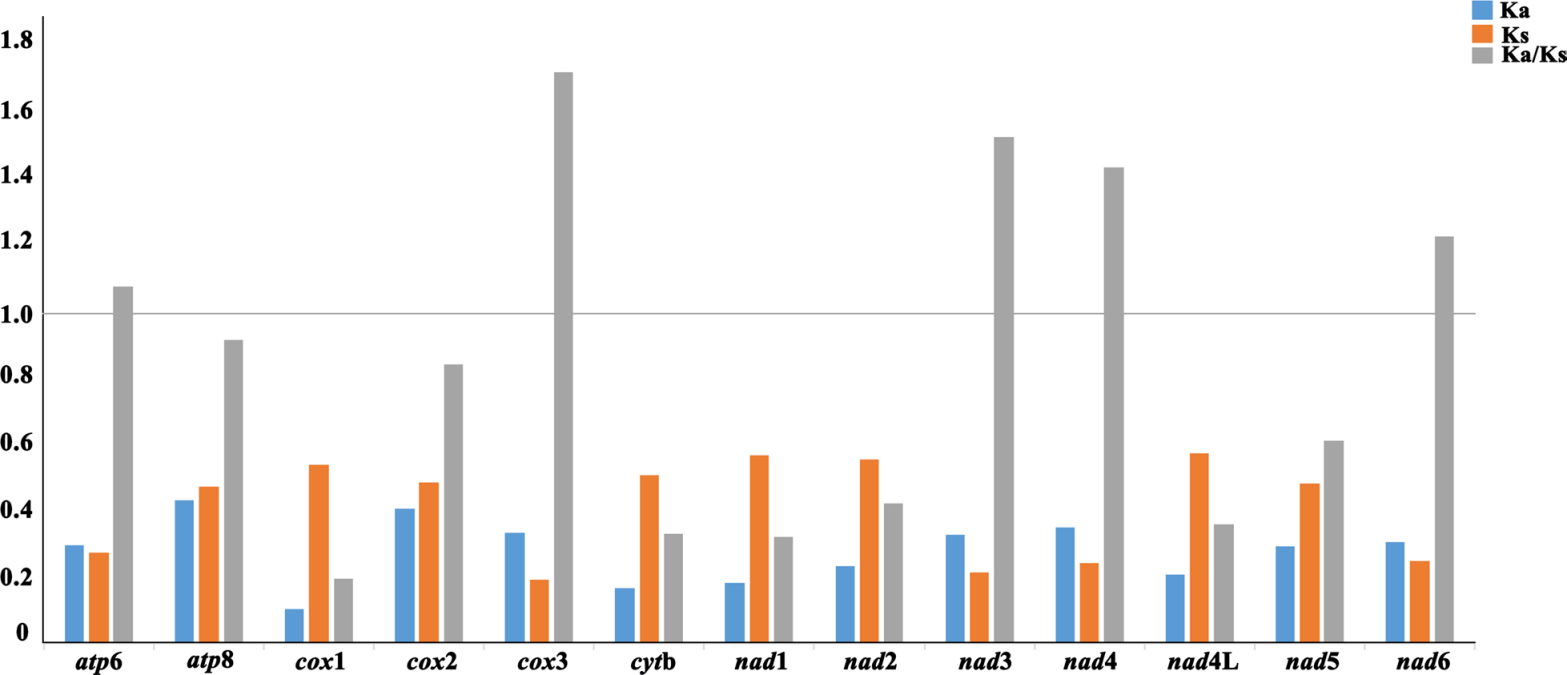
Substitution ratios in the mitochondrial genomes of capillariid nematodes. The rate of non-synonymous (Ka), the rate of synonymous (Ks) substitutions and the respective ratios (Ka/Ks) for individual protein-coding genes are shown

In PCGs, Ka/Ks test was helpful for better understanding the selective constraints, evolutionary changes, sexual selection and disease resistance [41]. Generally, the ratio of Ka/Ks being > 1 represents rapid evolution of a protein gene and a positive selection and changes in protein’s functions. In contrast, < 1 indicates a negative selection and selectively purified among genes [42, 43]. In the present study, the Ka/Ks ratios of the genes *atp*6, *cox*3, *nad*3, *nad*4 and *nad*6 ranged from 1.07 to 1.72 (Fig. 2), indicating these genes had experienced a positive selection or had undergone functional changes. The Ka/Ks values of *atp*8, *cox*1, *cox*2, *cyt*b, *nad*1, *nad*2, *nad*4L and *nad*5 were < 1 (Fig. 2), suggesting they were highly constrained within the family Capillariidae. Consistent with previous reports, the findings also indicated that *cox*1 was selectively purified and might play an adaptive role in the evolutionary process of Capillariidae species, and it can be used as useful marker to identify and distinguish capillariid species. Interestingly, *atp*8 gene was not only found in those species, but under purifying selection across Capillariidae, though it was variable protein-gene or even missing in some nematodes [44].

### Transfer and ribosomal RNA genes

**Fig. 3:**
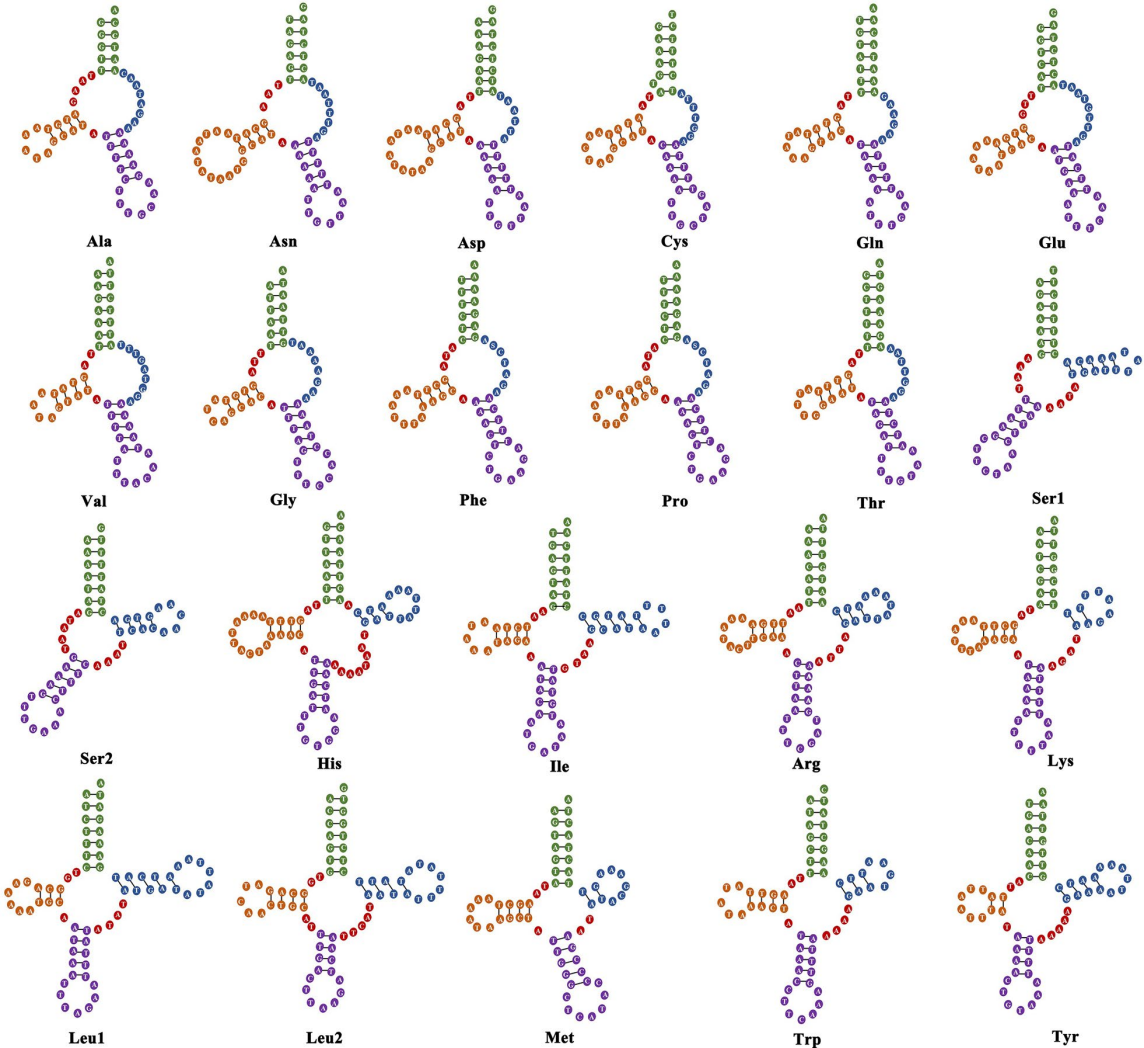
The secondary structures of 22 tRNA of *Aonchotheca putorii* mitochondrial genome. Eleven tRNAs have a typical TV-loop and nine tRNAs relatively standard “cloverleaf” structures; Leu1 and Leu2 for codon families CUN and UUR, respectively; S1 and S2 for codon families UCN and AGN, respectively

All 22 tRNAs were obtained in the present study ranging from 54 to 71 bp in length. The predicted secondary structures of most tRNAs identified in this study were similar to those found in other nematodes, and the TΨC stem loop and variable loop were replaced by a TV-replacement loop structure, which was common in the mitogenomes of nematodes excluding *Trichinella spiralis* (Owen, 1835) Railliet, 1895 [44–46]. Consistent with previous reports, the predicted structure of tRNA-Ser (AGN and UCN) lacked the DHU-arm, which was replaced by 4–5 nucleotide residues [47, 48]. In the present study, 9 of 20 tRNAs (tRNA-His, tRNA-Ile, tRNA-Arg, tRNA-Lys, tRNA-Leu^CUN^, tRNA-Leu^UUR^, tRNA-Met, tRNA-Trp, and tRNA-Tyr) presented relatively standard “cloverleaf” secondary structures like those of *T. spiralis* [44] (Fig. 3). The length of nine TΨC stems was from 2 to 5 bp, and the length of variable arms between the anticodon loop and TΨC stem-loop ranged from 2 to 8 bp.

The locations and boundaries of *rrn*L (16S rRNA) and *rrn*S (12S rRNA) were identified by alignment with available Capillariidae species (NC_056391). The typical arrangement of the *rrn*L was located between tRNA-Val and *atp*6, and the *rrn*S was situated in tRNA-Ser^AGN^ and tRNA-Val, similar to *Capillaria* sp. (MH665363), *E. annulatus* (MW999680) [33] and *P. tomentosa* (MZ708958). The length of these two rRNAs was 864 bp and 693 bp, respectively (Table 2).

### Comparative analyses of mitochondrial sequence and nuclear 18S rRNA

Comparisons of nucleotide sequences of mt genes are listed in Table 5, as were amino acids of PCG. Transfer RNA genes were not included in the table because *Capillaria* sp. missed four of them (tRNA-Tyr, tRNA-His, tRNA-Gly and tRNA-Cys). Obvious differences at both nucleotide and amino acid sequences were observed in all 13 PCG. Specifically, the differences of nucleotide and amino acid between *A. putorii* and *Capillaria* sp., *A. putorii* and *E. annulatus*, and *A. putorii* and *P. tomentosa* were 21.96–44.37%, 23.39–48.23% and 17.57–38.36% (Table 5) and 17.80–66.00%, 19.89–67.35% and 12.69–58.33%, respectively (Table 5). Furthermore, *cox*1 and *atp*8 were the most and the least conserved PCG, respectively. The differences of rRNAs nucleotide sequences were 18.61–30.99% in *rrn*L and 22.03–34.15% in *rrn*S (Table 5). It is acceptable that divergence of mt DNA sequences between species in nematodes was 10–20% [49]. The differences observed in the current study in nucleotide sequences of all 13 PCG and two rRNA genes ranged from 17.57 to 48.23%, and the divergences in amino acids were from 12.69 to 67.35% clearly indicated *A. putorii* was a distinct species from other Capillariidae species. The differences among *Aonchotheca*, *Capillaria*, *Eucoleus*, and *Pseudocapillaria* were higher than species level and further suggested *Aonchotheca* was a distinct genus.

**Table 5:** Differences in mitochondrial nucleotide and predicted amino acid sequences between Capillariidae species

Gene/region	NT^a^ size (bp)				NT^a^ difference (%)			Number of AA^b^				AA^b^ difference (%)		
	Ap	Cs	Ea	Pt	Ap/Cs	Ap/Ea	Ap/Pt	Ap	Cs	Ea	Pt	Ap/Cs	Ap/Ea	Ap/Pt
*atp*6	909	840	789	798	32.45	35.59	24.16	302	279	262	265	44.88	56.44	34.98
*atp*8	146	153	141	147	44.37	48.23	38.36	48	50	46	48	66.00	67.35	58.33
*cox*1	1584	1548	1548	1548	21.96	23.39	17.57	527	515	515	515	17.80	19.89	12.69
*cox*2	684	684	684	681	28.05	31.39	23.64	227	227	227	226	30.26	34.65	23.25
*cox*3	774	774	777	774	31.78	33.59	25.07	257	257	258	257	36.82	40.93	26.74
*cyt*b	1136	1113	1113	1113	25.36	27.07	21.31	378	370	370	370	30.42	31.75	24.34
*nad*1	912	894	900	894	29.87	31.11	24.16	303	297	299	297	36.30	36.30	23.76
*nad*2	900	603	903	900	33.83	37.12	24.23	299	200	300	299	63.00	54.82	27.67
*nad*3	301	342	330	342	32.20	35.36	19.86	100	113	109	113	48.28	59.48	34.48
*nad*4	1263	1218	954	1260	36.95	35.01	25.16	420	1218	317	419	51.78	60.81	30.10
*nad*4L	249	249	237	249	33.34	35.87	28.93	82	82	78	82	43.37	45.12	32.93
*nad*5	1521	1560	1560	1542	40.26	39.54	25.25	506	519	519	513	51.25	51.15	28.41
*nad*6	459	459	461	456	32.24	35.79	25.00	152	152	153	151	48.70	50.97	35.29
*rnn*L	864	902	842	941	27.98	30.99	18.61	–	–	–	–	–	–	–
*rnn*S	693	701	814	676	32.05	34.15	22.03	–	–	–	–	–	–	–

*Ap Aonchotheca putorii*, *Cs Capillaria* sp., *Ea Eucoleus annulatus*, *Pt Pseudocapillaria tomentosa*

^a^Nucleotide

^b^Amino acid

### Phylogenetic analyses

BI and ML trees of 18S rRNAs of Capillariidae species (Additional file 2: Figure S1) showed similar topologies to previous studies [5, 50]. These indicated some *Capillaria* species did not belong to this genus. The tree further proved that genera *Capillaria* and *Eucoleus* were all monophyly and more related, the genus *Aonchotheca* might be paraphyly, and results also showed a closer relationship among genera *Aonchotheca*, *Calodium*, *Pearsonema*, *Pseudocapillaria*, and *Baruscapillaria* with moderate supports (Additional file 3: Figure S2).

**Fig. 4:**
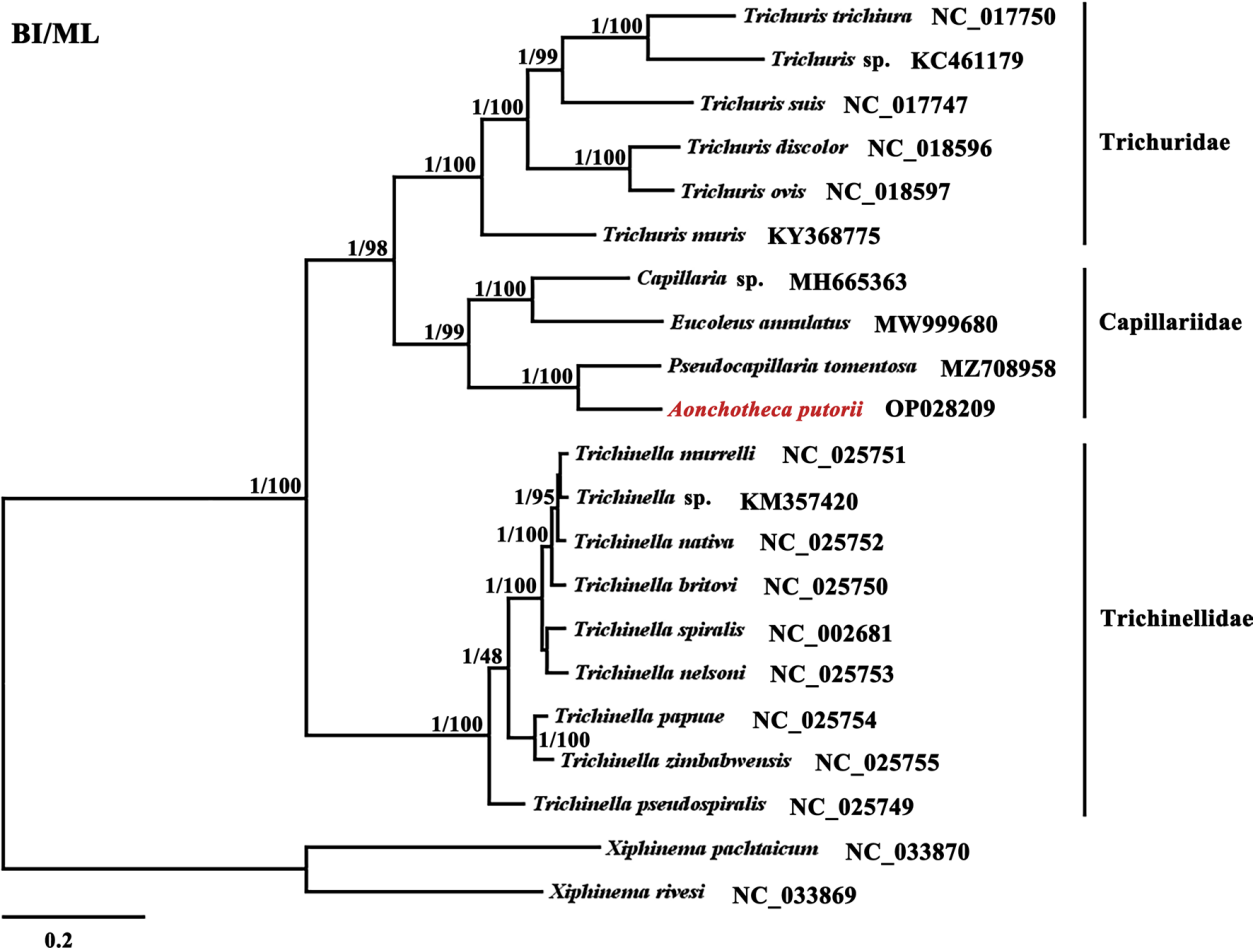
Phylogenetic relationships among 18 species of Trichinellida nematodes inferred from Bayesian and maximum likelihood analyses of deduced amino acid sequences of 12 mitochondrial proteins. *Xiphinema pachtaicum* (GenBank no. NC_033870) and *Xiphinema rivesi* (GenBank no. NC_033869) were used as outgroups. Bayesian posterior probability (Bpp) and bootstrap frequency (Bf) values were indicated at nodes. The former of the phylogenetic tree represents parasitic nematode, and the latter represents their hosts. Most Trichinellidae strain species were maintained by serial passage in female CD1 mice, and they were not labeled in the figure

Using *X. pachtaicum* and *X. rivesi* as the outgroups in analyzing phylogenetic relationships within class Trichinellida, both BI and ML trees displayed similar topological and systematic relationships (Fig. 4), which was consistent with previous data [33, 51]. The families Capillariidae, Trichinellidae and Trichuridae were clearly in their own clades. In accord with previous studies based on complete mitogenome or 18S rRNA sequences [33, 50, 51], our results also showed those families were monophyletic with Capillariidae and Trichuridae forming sister taxa of high statistical support (Bpp = 1, Bf ≥ 98).

In the family Capillariidae, *Capillaria* was a sister taxon genus to *Eucoleus*, *Pseudocapillaria* and *Aonchotheca*, of high support (Bpp = 1, Bf ≥ 99). Furthermore, there was a closer relationship between genera *Aonchotheca* and *Pseudocapillaria*, and between *Capillaria* and *Eucoleus*, which was similar to the previous results by analyzing *cox*1 and 18S rRNA [4, 5, 50, 51], indicating *cox*1 and 18S rRNA genes were useful markers to mark capillariids. In addition, the distances between the *Capillaria* species (*Capillaria* sp.) and the other three capillariid nematodes (*E*. *annulates*, *P*. *tomentosa* and *A*. *putorii*) were longer than the distances within other trichinellid species, which further clarified long genetic distance among those genera. These phylogenetic results also indicated that *Eucoleus*, *Pseudocapillaria*, and *Aonchotheca* were three distinct genera from *Capillaria*, supporting the accuracy of Moravec taxonomic revision [3, 5, 50]. However, there are still taxonomic controversies within Capillariidae concerning phylogenomics and systematic status [4, 19, 33] due to the limited numbers of complete mitogenome in other Capillariidae species, like *Baruscapillaria*, *Calodium*, *Paracapillaria* and *Pearsonema*.

## Conclusion

We have revealed details of the mitogenome of *A*. *putorii* and showed that *Aonchotheca* is a distinct genus from *Capillaria*. Our data indicate mitogenome is an ideal tool for analyzing the phylogenetic relationships and dealing with systematic controversy. In addition, nuclear DNA of 18S rRNA agrees with the phylogenetic results of mitogenome. Though the molecular information in capillariids has steadily increased recently, mt DNA sequences are still in great demand to reveal evolutionary rate, phylogenomic and systematic relationships within this family.

## Supplementary Information

**Additional file 1: Table S1.** The designed primers were used to amplify the complete mitochondrial sequence of *Aonchotheca putorii*. **Table S2** The uncorrected paired genetic distance of 18S rRNAs among the Capillariidae species. **Table S3** The uncorrected paired genetic distance of 18S rRNAs among the *Aonchotheca putorii *from different regions.

**Additional file 2: Figure S1.** The electrophoretogram of the verified complete mitochondrial genome of *Aonchotheca putorii*. M: 5000 marker; -: negative control; Lane 1: tRNA-Gly – nad4; Lane 2: nad4 – nad4L; Lane 3: rrnS – atp8; Lane 4: atp8 – nad1; Lane 5: nad4L – rrnS.

**Additional file 3. Figure S2.** The phylogenetic analyses based on 18S rRNA among the family Capillariidae using Bayesian posterior probability (Bpp) and Bootstrap frequency (Bf) values were indicated at nodes. The former of the phylogenetic tree represents parasitic nematodes, and the latter represents their hosts.

## Data Availability

The complete mitochondrial genome and 18S rRNA sequences of *Aonchotheca putorii* from the hedgehogs have been deposited in the GenBank under the accession numbers OP028951 and OP028209, respectively.
