# Supplementary material for: Aonchotheca (Nematoda: Capillariidae) is validated as a separated genus from Capillaria by both mitochondrial and nuclear ribosomal DNA
Source: Parasit Vectors. 2022 Dec 30;15:493. doi: 10.1186/s13071-022-05609-9 (PMC9805247; doi:10.1186/s13071-022-05609-9)
Supplement: Supplementary file 1 — Additional file 1: Table S1. The designed primers were used to amplify the complete mitochondrial sequence of Aonchotheca putorii. Table S2 The uncorrected paired genetic distance of 18S rRNAs among the Capillariidae species. Table S3 The uncorrected paired genetic distance of 18S rRNAs among the Aonchotheca putorii from different regions. [file 13071_2022_5609_MOESM1_ESM.docx]

**Table S1** The designed primers were used to amplify the complete mitochondrial sequence of *Aonchotheca putorii*.

| **Name** | **Sequences (5’-3’)** | **Length (bp)** |
| --- | --- | --- |
| CWF1 | ACTAAATGTGCTGATACACTAAAT | ~ 2,900 |
| CWR1 | TTGGATGAGGAAATCAACCAGAACGG |  |
| CWF2 | TTATCCGTTCTGGTTGATTTCCTC | ~ 1,500 |
| CWR2 | TAATAATTCGTCAACACGGTAA |  |
| CWF3 | CTCGTAAGAGGTAAGTCGTAACAA | ~ 3,100 |
| CWR3 | ATAAACTAAAGGACTAACTTGATA |  |
| CWF4 | TATCAAGTTAGTCCTTTAGTTTAT | ~ 3,900 |
| CWR4 | ATATCGGACTCGTGGCAGTGTATA |  |
| CWF5 | CAATGATTCTATGAACTACTCTTA | ~ 2,700 |
| CWR5 | TCTACTTTGTTACGACTTACCTCT |  |

**Table S2** The uncorrected paired genetic distance of 18S rRNAs among the Capillariidae species.

| **Species/Divergence (%)** | **1** | **2** | **3** | **4** | **5** | **6** | **7** | **8** | **9** | **10** | **11** | **12** | **13** | **14** | **15** | **16** | **17** | **18** | **19** |
| --- | --- | --- | --- | --- | --- | --- | --- | --- | --- | --- | --- | --- | --- | --- | --- | --- | --- | --- | --- |
| *Baruscapillaria obsignata* |  |  |  |  |  |  |  |  |  |  |  |  |  |  |  |  |  |  |  |
| *Aonchotheca riukiuensis* | 3.2 |  |  |  |  |  |  |  |  |  |  |  |  |  |  |  |  |  |  |
| *Capillaria suis* | 3.6 | 1.3 |  |  |  |  |  |  |  |  |  |  |  |  |  |  |  |  |  |
| *Calodium hepaticum* | 3.0 | 1.3 | 1.7 |  |  |  |  |  |  |  |  |  |  |  |  |  |  |  |  |
| *Capillaria bursata* | 3.3 | 2.0 | 2.2 | 1.8 |  |  |  |  |  |  |  |  |  |  |  |  |  |  |  |
| *Aonchotheca* sp. | 3.1 | 1.6 | 1.8 | 1.3 | 1.7 |  |  |  |  |  |  |  |  |  |  |  |  |  |  |
| *Pearsonema* sp. | 2.6 | 1.3 | 1.7 | 1.0 | 1.7 | 1.1 |  |  |  |  |  |  |  |  |  |  |  |  |  |
| *Aonchotheca musimon* | 3.0 | 2.0 | 1.9 | 1.4 | 2.2 | 1.7 | 1.1 |  |  |  |  |  |  |  |  |  |  |  |  |
| *Aonchotheca putorii* (Japan) | 2.6 | 1.7 | 2.2 | 1.5 | 1.9 | 1.5 | 1.1 | 1.5 |  |  |  |  |  |  |  |  |  |  |  |
| *Aonchotheca putorii* (China) | 2.6 | 1.7 | 2.2 | 1.5 | 1.9 | 1.5 | 1.1 | 1.5 | 0 |  |  |  |  |  |  |  |  |  |  |
| *Aonchotheca paranalis* | 3.0 | 2.0 | 2.4 | 1.7 | 2.5 | 2.1 | 1.4 | 1.9 | 1.8 | 1.8 |  |  |  |  |  |  |  |  |  |
| *Pseudocapillaria tomentosa* | 3.8 | 3.4 | 3.4 | 3.1 | 3.5 | 3.3 | 3.0 | 3.2 | 3.3 | 3.3 | 3.3 |  |  |  |  |  |  |  |  |
| *Eucoleus contortus* | 9.4 | 9.3 | 9.9 | 9.4 | 10.1 | 9.5 | 9.4 | 9.3 | 9.1 | 9.1 | 9.5 | 9.9 |  |  |  |  |  |  |  |
| *Eucoleus garfiai* | 9.7 | 9.5 | 10.1 | 9.7 | 10.5 | 9.9 | 9.8 | 9.6 | 9.5 | 9.5 | 9.9 | 10.2 | 1.1 |  |  |  |  |  |  |
| *Eucoleus perforans* | 10.3 | 10.3 | 10.4 | 10.1 | 10.9 | 10.1 | 10.1 | 9.7 | 10.1 | 10.1 | 10.4 | 10.6 | 2.5 | 2.2 |  |  |  |  |  |
| *Capillaria spinulosa* | 11.4 | 11.1 | 11.5 | 11.2 | 11.6 | 11.3 | 10.9 | 11.4 | 11.2 | 11.2 | 11.4 | 11.9 | 13.5 | 13.6 | 14.1 |  |  |  |  |
| *Capillaria pudendotecta* | 10.9 | 10.8 | 11.4 | 11.2 | 11.7 | 11.2 | 10.8 | 11.0 | 11.2 | 11.2 | 11.2 | 11.4 | 13.0 | 13.2 | 13.6 | 2.6 |  |  |  |
| *Capillaria anatis* | 9.0 | 9.1 | 9.4 | 9.1 | 9.6 | 9.1 | 9.1 | 9.3 | 9.1 | 9.1 | 9.2 | 10.1 | 11.8 | 12.1 | 12.7 | 10.2 | 9.5 |  |  |
| *Capillaria madseni* | 9.4 | 9.1 | 9.5 | 9.0 | 9.3 | 8.9 | 9.1 | 9.4 | 8.9 | 8.9 | 9.2 | 10.2 | 12.0 | 12.3 | 12.8 | 10.7 | 11.0 | 7.7 |  |
| *Capillaria tenuissima* | 10.9 | 10.6 | 11.1 | 10.7 | 11.4 | 10.8 | 10.7 | 10.8 | 10.8 | 10.8 | 10.6 | 11.6 | 12.3 | 12.4 | 13.4 | 12.0 | 11.8 | 10.4 | 10.0 |

**Table S3** The uncorrected paired genetic distance of 18S rRNAs among the *Aonchotheca putorii* from different regions.

| ***A. putorii* Species**  **/Divergency (%)** | **1** | **2** | **3** | **4** | **5** | **6** | **7** | **8** |
| --- | --- | --- | --- | --- | --- | --- | --- | --- |
| LC052353 Kyoto |  |  |  |  |  |  |  |  |
| LC052354 Wakayama | 0 |  |  |  |  |  |  |  |
| LC052356 Saga | 0.22 | 0.22 |  |  |  |  |  |  |
| LC052349 Shizuoka | 0.67 | 0.67 | 0.84 |  |  |  |  |  |
| *A. putorii* China | 0.67 | 0.67 | 0.84 | 0 |  |  |  |  |
| LC052364 Kochi | 0.61 | 0.61 | 0.78 | 0.17 | 0.17 |  |  |  |
| LC052350 Shimanto | 0.56 | 0.56 | 0.72 | 0.11 | 0.11 | 0.06 |  |  |
| LC052357 Nagasaki | 0.56 | 0.56 | 0.72 | 0.11 | 0.11 | 0.06 | 0 |  |
| LC052361 Shiga | 0.78 | 0.78 | 0.89 | 0.89 | 0.89 | 0.83 | 0.78 | 0.78 |
