# Supplementary figures and images for: Aonchotheca (Nematoda: Capillariidae) is validated as a separated genus from Capillaria by both mitochondrial and nuclear ribosomal DNA
Source: Parasit Vectors. 2022 Dec 30;15:493. doi: 10.1186/s13071-022-05609-9 (PMC9805247; doi:10.1186/s13071-022-05609-9)

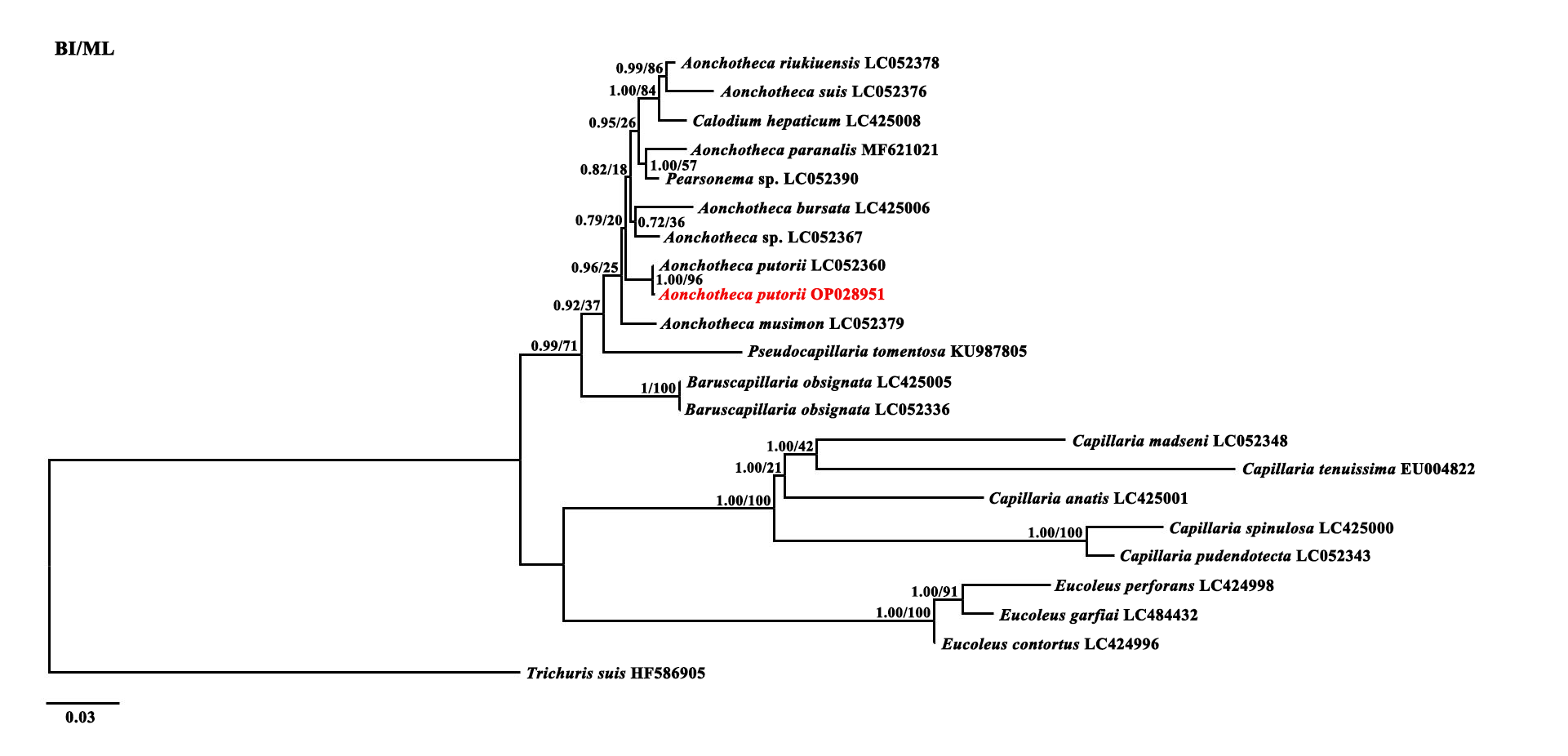

Supplement: Supplementary file 3 — Additional file 3. Figure S2. The phylogenetic analyses based on 18S rRNA among the family Capillariidae using Bayesian posterior probability (Bpp) and Bootstrap frequency (Bf) values were indicated at nodes. The former of the phylogenetic tree represents parasitic nematodes, and the latter represents their hosts. [file 13071_2022_5609_MOESM3_ESM.tif]
